# The influence of MRI-based pelvimetric measurements in mother’s choice of delivery in fetal breech position

**DOI:** 10.1007/s00404-023-07348-3

**Published:** 2024-02-09

**Authors:** Anna Elisabeth Ebeling, Sabine Katharina Maschke, Sophia Holthausen-Markou, Lena Steinkasserer, Rüdiger Klapdor, Diane Renz, Nina Meier, Constantin von Kaisenberg, Peter Hillemanns, Lars Brodowski

**Affiliations:** 1 Department of Obstetrics, Gynecology and Reproductive Medicine, Hannover Medical School, Carl-Neuberg-Str. 1, 30625 Hannover, Germany; 2 Department of Diagnostic and Interventional Radiology, Hannover Medical School, Hannover, Germany

**Keywords:** Breech position, Shared decision-making, Pregnancy

## Abstract

**Introduction:**

At term, about 3–4% of all singleton pregnancies present as breech. MRI-based pelvimetry is a valuable tool to support selection of adequate candidates for a trial-of-labor in women expecting term breech babies. Shared decision-making is playing an increasingly important role in obstetrics. Since the divergent existing knowledge of breech term delivery needs to be discussed with the pregnant woman, we examined the influence of MRI results on the shared decision-making process in women with term breech presentation.

**Methods:**

Between 08/2021 and 12/2022, anamnestic and clinical parameters were collected from singleton pregnancies expecting term breech babies resulting in birth at the Hanover Medical School. After information, written consent and inclusion, clinical parameters, the course of birth and the maternal and fetal outcome were collected retrospectively. 32 women participated in a postpartum questionnaire study on inquiry. The subsequent acquisition of information and the arguments in the decision-making process were determined. In addition, the sense of security and self-determination was asked both before and during birth.

**Results:**

50% of the respondents had not decided for a mode of delivery before having MRI pelvimetry. After imaging and information, about the own pelvic dimensions and predictors for a successful vaginal birth, 80% of this subgroup decided to give birth vaginally. Over 40% of the collective descripted that they made a decision based on the result of MRI pelvimetry. None of the women felt to be insecure after having talked about the MRI results. The elective cesarean section group and the group of those who delivered vaginally were approximately equally highly satisfied with their feeling of self-determination of the birth mode. Overall, the study population had a very positive birth experience. The group of women who had delivered by elective cesarean showed a wider range in their assessment and appeared to perceive the experience more negative than the group of women who had a vaginal birth or emergency cesarean. Fetal and maternal outcomes did not differ between the groups.

**Discussion:**

MRT pelvimetry measurements can be used as a predictor for a successful vaginal breech delivery. The additional information obtained from the MRI measurements can be used in the shared decision-making process to decide more easily on the mode of delivery while improving women's awareness and safety. A balanced education on rare and frequently adverse events of vaginal delivery and cesarean section and patient expectations about labor processes must be taken into account.

## What does this study add to the clinical work

**Table:** 

MRI pelvimetry can support the shared decision making process as a tool in birth mode planning for breech presentation. The method can provide further security for choosing a mode of birth; uncertainty was not observed.	

## Introduction

At term, about 3–4% of all singleton pregnancies present as breech. Breech presentation is a physiological phenomenon during pregnancy, and its persistence toward the end of pregnancy can have various causes, such as uterine malformations [1]. However, in many cases, there is no identifiable underlying cause.

The mode of delivery has been discussed intensively in professionals for a long time. There has been a lack of studies on this subject, which led to uncertainty among obstetricians. The multicentric, randomized controlled study of Hannah et al. concluded that vaginal breech delivery leads to significantly higher fetal morbidity and mortality. Thus, the decision for an elective cesarean section was preferable to vaginal delivery [2]. After the study was published, there was a significant increase in the number of cesarean sections worldwide in response to the diagnosis of breech presentation. A survey in 2003 covering 80 countries showed that a planned cesarean section was aimed in 92.5% of all breech cases [3]. However, criticism arose that the results of the Term Breech Trial were not tenable and that the reproducibility was only conditional due to the collective selected. There was lack of uniform examination criteria and expertise of experienced obstetricians during the implementation, which is why the vaginal deliveries were sometimes unsuccessful [4]. In addition, the fetal long-term outcome was not considered. Depending on the country of birth, 69–100% of all breech births are by cesarean section. According the statement of the World Health Organization (WHO), only about 15% of all cesarean sections are medically indicated. Analyses of the neonatal long-term outcome show a significantly increased risk of obesity, respiratory system infections and asthma in surgically delivered children [5]. A higher risk of developing neurological diseases and type 1 diabetes mellitus is also discussed [5]. It has been shown that under certain conditions, vaginal delivery is not worse in long-term fetal outcome compared to cesarean section [6]. In addition, the cesarean section leads to higher maternal morbidity [7].

Crucially, the wishes of the pregnant women regarding the mode of delivery need to be considered. Birth mode decision-making is very individual and shaped by the cultural, the spiritual, and the personal experiences of the women. In many cases, the diagnosis of breech presentation immediately leads to uncertainty [8]. It is important that the patient receives information from the physician about the available options and is informed about the advantages as well as the risks. The aim is to find a joint decision, while maintaining the autonomy of the person seeking advice. This proven approach has also found its way into the local obstetrical guidelines, which applies to the collective examined here [9].

Referring the term breech trial, a particular patient population does not benefit from vaginal delivery due to higher fetal short-term risks. The goal is to identify the group that does not meet certain risk factors and therefore would benefit from a spontaneous birth. The MRI pelvimetry can provide further information of maternal pelvic structures to assess a successful vaginal delivery attempt. MRI-based pelvimetry is a valuable tool to support selection of adequate candidates for a trial-of-labor in women expecting term breech babies. Shared decision-making (SDM) between patients and healthcare providers is a model for making patient-centered healthcare decisions and achieving value-based care [10–13]. SDM is a process of defining a goal, checking the individual requirements, and discussing the available options [14]. The beginning of the process is the identification of a goal—here the intended mode of delivery in fetal breech position. The healthcare professionals are the experts of the medical evidence, whereas the patients are the experts on what matters the most to them [12].

The divergent existing knowledge of breech term delivery needs to be discussed with the pregnant woman. In this study, we examined the influence of MRI pelvimetry results on the shared decision-making process in women with term breech presentation.

## Methods

### Study design

This is an analytical semi-longitudinal observational study. All pregnant women with a breech presentation were counseled between 34 and 37 weeks of gestation. External cephalic version, vaginal attempted birth, as well as cesarean delivery were discussed with each patient, depending on the individual patient history and examination. Inclusion criteria for a vaginal attempted birth were an estimated fetal weight between 2500 and 4000 g and a gestational age of 36 + 0 weeks of pregnancy or higher. Exclusion criteria were language barriers, which did not allow participation in the questionnaire part of the study.

### Data collection

Between 08/2021 and 12/2022, anamnestic and clinical parameters were collected from singleton pregnancies expecting term breech babies resulting in birth at the Hanover Medical School.

A total of 3906 children were born in the clinic during this period. The general cesarean section rate was 30.3%. A total of 150 pregnant women with breech position presented to the clinic to plan birth. Of these, 20 pregnant women were recommended primary cesarean section due to a maternal or fetal indication. The birth mode was discussed with the remaining 130 women. 49 women decided to attempt spontaneous birth, 81 women opted for primary cesarean section.

We identified 41 patients who received MRI pelvimetry after being diagnosed with breech presentation.

After information, written consent and inclusion, clinical parameters, the course of birth and the maternal and fetal outcome were collected retrospectively. 32 of the 41 identified women participated in a postpartum questionnaire study on inquiry. The questions addressed information received from the obstetrician, knowledge, and concerns about delivery in breech position. The subsequent acquisition of information and the arguments in the decision-making process were determined. In addition, the sense of security and self-determination was asked both before and during birth.

### Assessment instruments

Obstetricians experienced in vaginal as well as cesarean breech deliveries should be involved in consultation in mode of delivery guided by the patient's individual risk factors. The counseling process in our collective was as follows:

All pregnant women with a breech presentation were counseled between 34 and 37 weeks of gestation. Mean gestational age of first presentation was 36.3 (SD 1.5) weeks of gestation. After examination and sonographic fetometry, various options of delivery mode were presented. Here, risks and complications of vaginal delivery and C-section were explained in detail. In addition, the possibility and the success rate of external version were explained, related to current literature and experience of the hospital. 37.5% of the study population took part in attempt of external cephalic version, 62.5% did not. After frustrating external cephalic version or non-participating, the study group was informed about the option of MRI pelvimetry. All women who did not immediately decide to go for cesarean section as mode of delivery after talking about options were offered an MRI scan independently of parity. While it was recommended for primiparous women, it was not strictly required for attempting a vaginal birth.

The collective was informed about the MRI as a non-invasive cross-sectional imaging method with no exposure to radiation, which does not endanger the safety of the fetus and mother [10]. All patients underwent MRI at 1.5 T (Avanto, Siemens Healthineers, Erlangen, Germany). The studies were planned and conducted according to the recommendations of the American College of Radiology for the safe and optimal performance of fetal MRI [15]. Axial and sagittal views were obtained to measure maternal pelvimetry as well as a high-resolution axial view to measure the fetal head using multiplanar reconstruction. Overall, the whole MRI scan took approximately 15 min. All patients were attended by a radiologist and a radiographer prior, during and after the examination. All patients received ear protection during the scan and held a bell to alert the technician in case of emergency. An additional blanket was offered to minimize the acoustic noise perceived by the fetus.

After receiving the MRI results, the available measurements of the obstetric conjugate, the intertuberous distance, and the pubic angle were compared with those in the literature and our own clinical experience to date. The mean values according to the literature were compared with the individual measured values of the pregnant women. Klemt et al. checked MRI-based pelvimetric measurements as predictors for a successful vaginal breech delivery in a collective of 633 nulliparous women. They found that the size of the obstetric conjugate correlates with rate of vaginal deliveries and can be seen as a pre-selectin criterion. They demanded an obstetric conjugate of 12 cm or greater. Although not significantly different, successful vaginal breech delivery could not be observed in women with an intertuberous distance of less than 10.9 cm and a pubic angle of less than 70°. This information was communicated to the study subjects [16].

If the obstetric conjugate was less than 11.5 cm, the intertuberous distance less than 10.9 cm or the pubic angle less than 70°, the pregnant woman was recommended to undergo primary cesarean section, as successful spontaneous parturition was not observed below these cut-offs either in the literature or in our own clinic. If the values were above these cut-offs, a vaginal delivery was offered.

The size of the obstetric conjugate and the intertuberous distance correlates with rate of vaginal deliveries [16]. This observation was explained to the pregnant women. It was emphasized that the MRI pelvimetry can provide further information but can only be seen in conjunction with other criteria, such as fetometry, fetomaternal Doppler, and the anamnesis of the pregnant woman. After discussing the MRI pelvimetry, vaginal attempted birth or cesarean delivery was planned.

After giving birth, data were collected by a questionnaire containing 25 questions, mostly exploratory multiple choice. Women answered the questions postpartum based on retrospective behaviors and perceptions during their recent pregnancy.

### Statistical analysis

Statistical analyses were performed using Graph pad prism 9 software (GraphPad Software Inc.). Means and standard deviation (SD) as well as numbers and percentages were calculated to present descriptive information. Statistical relevance was tested with Mann–Whitney *U*-test or unpaired *t*-test after determination of data distribution using the Shapiro–Wilk normality test.

### Ethics

This study was approved by the local ethics committee of the Hannover Medical School and conducted according to the principles of the Declaration of Helsinki (approval number: 10646_BO_K_2022). Informed consent was waived by the ethics committee due to the use of retrospective and de-identified data. However, patients who completed the postpartum questionnaire part were required to give written informed consent.

## Results

### Participants

After collecting the clinical parameters, all participants (*N* = 32) were asked to participate in answering the postpartum questionnaire. The mean age at time of delivery was 32.2 years (SD 3.7), the number of primiparous was 26 (81.3%), and maternal gestational age at birth was 40.2 weeks (SD 0.7). 23 (71.9%) women delivered vaginal, 9 (28.1%) women delivered via cesarean section while 5 (15.6%) had an elective and 4 (12.5%) an emergency cesarean section. The majority, 19 individuals (82.6%) of vaginal deliveries were primiparous. Four multiparous had vaginal birth (17.4%). Seven primiparous had given birth by cesarean. While three of those women had chosen elective cesarean, four women had to undergo emergency cesarean. Only two multiparous had a C-section, which was elective in both cases.

Most participants had a high education, were married or in an informal relationship and native speaker. The most common pregnancy complication was a gestational diabetes with 6.3% of the study group, and cervical insufficiency and mild fetal malformation were rare (Table 1).

**Table 1:** Structure of the study group

Characteristics	Study population	Vaginal delivery	Cesarean delivery	Elective cesarean	Emergency cesarean
Number of women, *N* (%)	32 (100)	23 (71.9)	9 (28.1)	5 (15.6)	4 (12.5)
Maternal age, mean ± STD	32.2 ± 3.7	31.6 ± 3.6	33.8 ± 3.5	36.4 ± 1.5	30.5 ± 2.1
Pre-pregnancy BMI, mean ± STD	22.9 ± 3.8	23.3 ± 4.1	22.0 ± 3.0	21.3 ± 2.3	23.2 ± 4.1
Primiparous, *N* (%)	26 (81.3)	19 (82,6)	7 (77.8)	3 (60)	4 (100)
Multiparous, *N* (%)	6 (18.8)	4 (17.4)	2 (22.2)	2 (40)	0 (0)
Education, *N* (%)					
Higher	25 (78.1)	18 (78.3)	7 (77.8)	4 (80)	3 (75)
Vocational	5 (15.6)	4 (17.4)	2 (22.2)	1 (20)	1 (25)
High school	2 (6.3)	1 (4.4)	0 (0)	0 (0)	0 (0)
Marital status, *N* (%)					
Married	22 (68.8)	17 (73.9)	5 (55.6)	3 (60)	2 (50)
Informal relationship	10 (31.3)	6 (26.1)	4 (44.4)	2 (40)	2 (50)
Native speaker, *N* (%)	30 (94)	22 (95.6)	8 (88.9)	5 (100)	3 (75)
Pregnancy complications, *N* (%)					
None	27 (84.4)	18 (78.3)	9 (100)	5 (100)	4 (100)
Gestational diabetes	2 (6.3)	2 (8.6)	0 (0)	0 (0)	0 (0)
Cervical insufficiency	1 (3.1)	1 (4.3)	0 (0)	0 (0)	0 (0)
Fetal malformation	2 (6.3)	2 (8.6)	0 (0)	0 (0)	0 (0)

### Timepoints of diagnosis and examination of breech position

The diagnosis of breech presentation was primarily made by the attending gynecologist as part of prenatal care. Only in one case, the diagnosis was made by the clinic. The mean gestational age at the time of diagnosis was 30.0 weeks of gestation (SD 4.1). On average, the women presented for birth mode planning at 36.3 weeks of gestation (SD 1.5). All individuals were offered to try external cephalic version. This was attempted in 12 cases (37.5%). With this not being successful, 11 of 12 women gave birth vaginally later. 20 participants decided against cephalic version. All five women who made the decision to have elective cesarean were part of this group. Until the final planning of the birth mode, there were an average of 3.9 (SD 1.4) consultations in the clinic. MRI pelvimetry was performed at 37.9 weeks of gestation (SD 0.8), while delivery occurred at 40.2 weeks of gestation (SD 0.7) in average (Table 2).

**Table 2:** Timepoints of diagnosis and examination of breech position

Characteristics	Study population	Vaginal delivery	Cesarean delivery	Elective cesarean	Emergency cesarean
Making diagnosis breech position, *N* (%):					
Gynecologist	31 (96.9)	23 (100)	8 (88.9)	4 (80)	4 (100)
Midwife	0 (0)	0 (0)	0 (0)	0 (0)	0 (0)
Clinic	1 (3.1)	0 (0)	1 (11.1)	1 (20)	0 (0)
Gestational age at getting diagnosis breech position, mean ± STD	30.0 ± 4.1	29.7 ± 4.2	30.7 ± 3.9	30.4 ± 5.5	31.0 ± 0.8
Gestational age at first presentation, mean ± STD	36.3 ± 1.5	36.4 ± 1.1	36.1 ± 2.3	36.0 ± 2.0	36.3 ± 2.9
Cephalic version					
Tried, *N* (%)	12 (37.5)	11 (47.8)	1 (11.1)	0 (0)	1 (25)
Not tried, *N* (%)	20 (62.5)	12 (52.2)	8 (88.9)	5 (100)	3 (75)
Amount of consultation until determination of mode of delivery, mean ± mean	3.9 ± 1.4	4.1 ± 1.5	3.6 ± 1.2	3.6 ± 0.9	3.5 ± 1.7
Gestational age at MRT examination, mean ± STD	37.9 ± 0.8	38.0 ± 0.8	37.7 ± 1.0	37.2 ± 1.1	38.3 ± 0.5
Gestational age at delivery, mean ± STD	40.2 ± 0.7	40.1 ± 0.6	40.3 ± 0.9	40.0 ± 0.7	40.8 ± 1.0

### Reaction on diagnosis of breech position

**Fig. 1:**
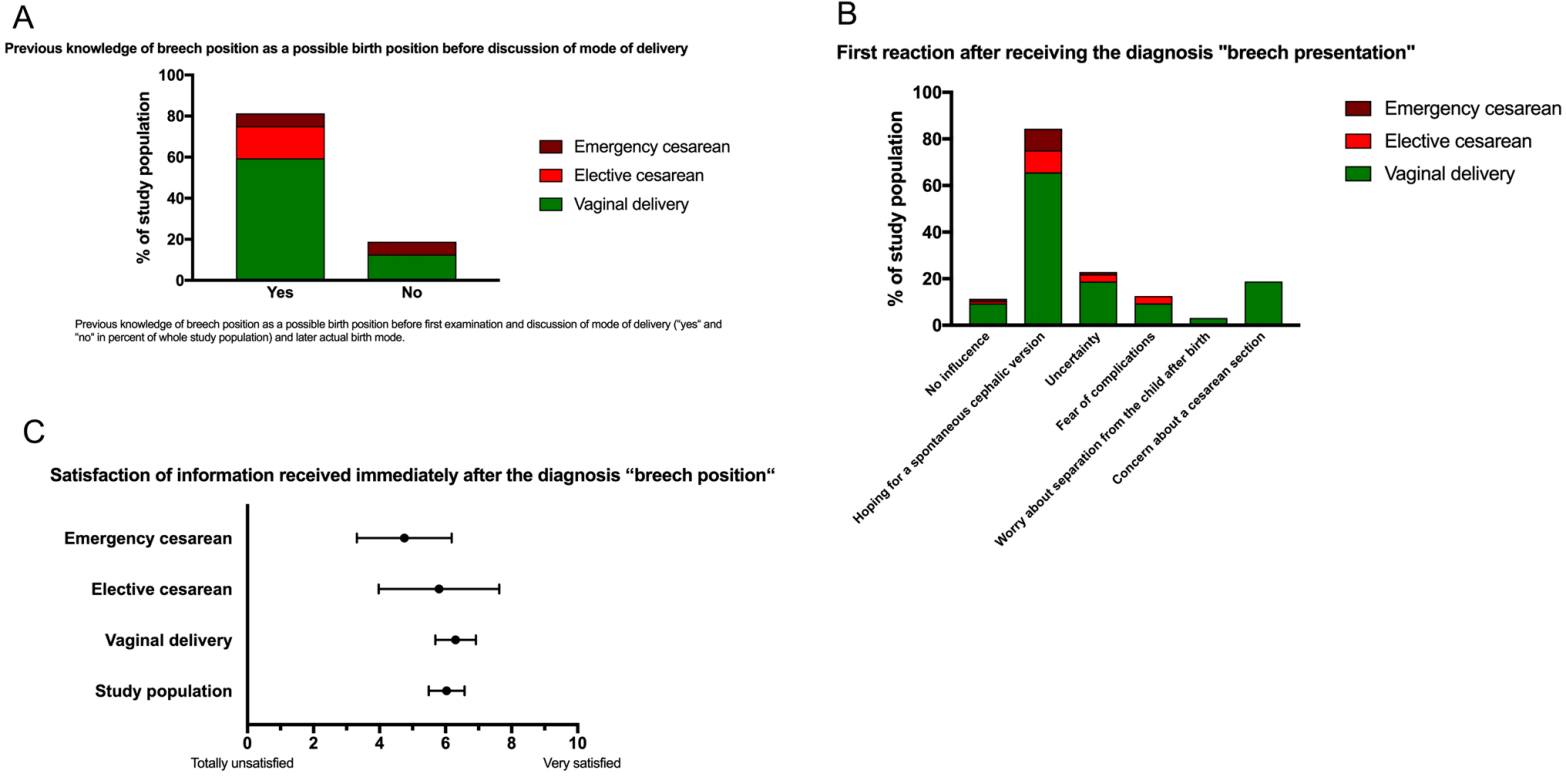
Previous knowledge of breech position, first reaction after receiving diagnosis and satisfaction of information received are shown. Fraction of collective with vaginal delivery is shown in green, elective cesarean is shown in red and emergency cesarean is shown in brown

26 women (81,25%) stated that they knew about breech position as a possible birth position. After receiving the information of breech position of their babies, approximately 80% of women felt the hope of a spontaneous cephalic version. 23% had felt uncertainty and 13% feared complications during pregnancy and childbirth. Only 10% were not influenced by the diagnosis (Fig. 1).

The satisfaction of received information immediately after the diagnosis of the study population was in a medium range and is shown in Fig. 1C.

### Process of decision-making

**Fig. 2:**
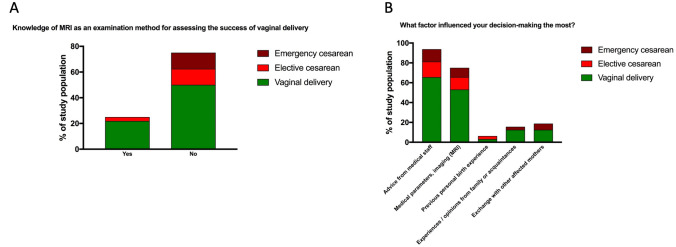
Knowledge of MRI as an examination method for assessing success of vaginal delivery and factors which influenced the decision process. Fraction of collective with vaginal delivery is shown in green, elective cesarean is shown in red and emergency cesarean is shown in brown

The collective described several sources of information apart from the consultation in the maternity hospital in order to make a decision regarding the mode of delivery. 72% of the women obtained information via the internet. 60% of the collective tried to get information from their midwife. Recommendations from family and friends as well as specialist literature were used by 40%. Utilization of the various sources of information was roughly evenly distributed across the delivery mode groups. The majority of the study group did not know about MRI pelvimetry as an examination method for assessing the success of vaginal delivery, but found it to be an important factor during decision-making regardless the later chosen birth mode, when asked retrospectively. Only the advice from medical staff influenced the study population more impressive. Personal previous experience, recommendations from friends or family, or reports from third parties had little influence on the choice of delivery mode (Fig. 2).

**Fig. 3:**
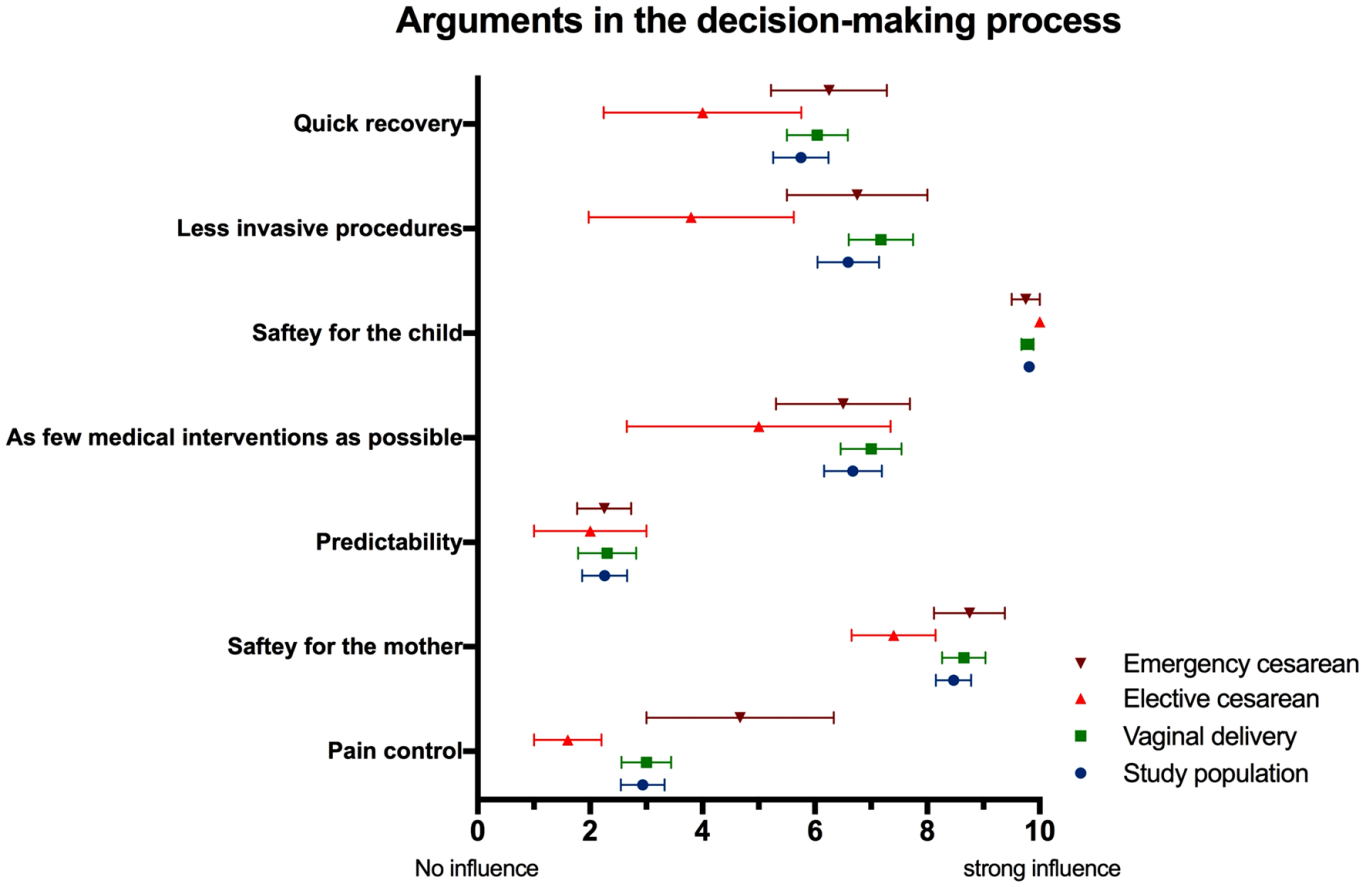
Arguments in decision-making process shown as a linear representation from no to maximum influence. Fraction of collective with vaginal delivery is shown in green, elective cesarean is shown in red and emergency cesarean is shown in brown

When asked to bring forward arguments for decision-making, safety for the child and the mother was most rated in all groups. Women who decided on having an elective cesarean evaluated the need of invasive procedures, medical interventions and a quick recovery as not as important in the decision-making process in comparison to the group of vaginal delivery. Predictability and pain control were weak arguments in all groups (Fig. 3).

### Influence of MRI pelvimetry in birth mode decision

50% of the respondents had not decided for a mode of delivery before having MRI pelvimetry. After imaging and information about the favorable pelvic dimensions and the possibility of a vaginal birth, 80% of this subgroup decided to give birth vaginally. Over 40% of the collective described that they made a decision based on the result of MRI pelvimetry, and 60% were confirmed in their decision by the MRI result, regardless of the planned type of birth mode. None of the women felt to be insecure after having talked about the MRI results.

**Fig. 4:**
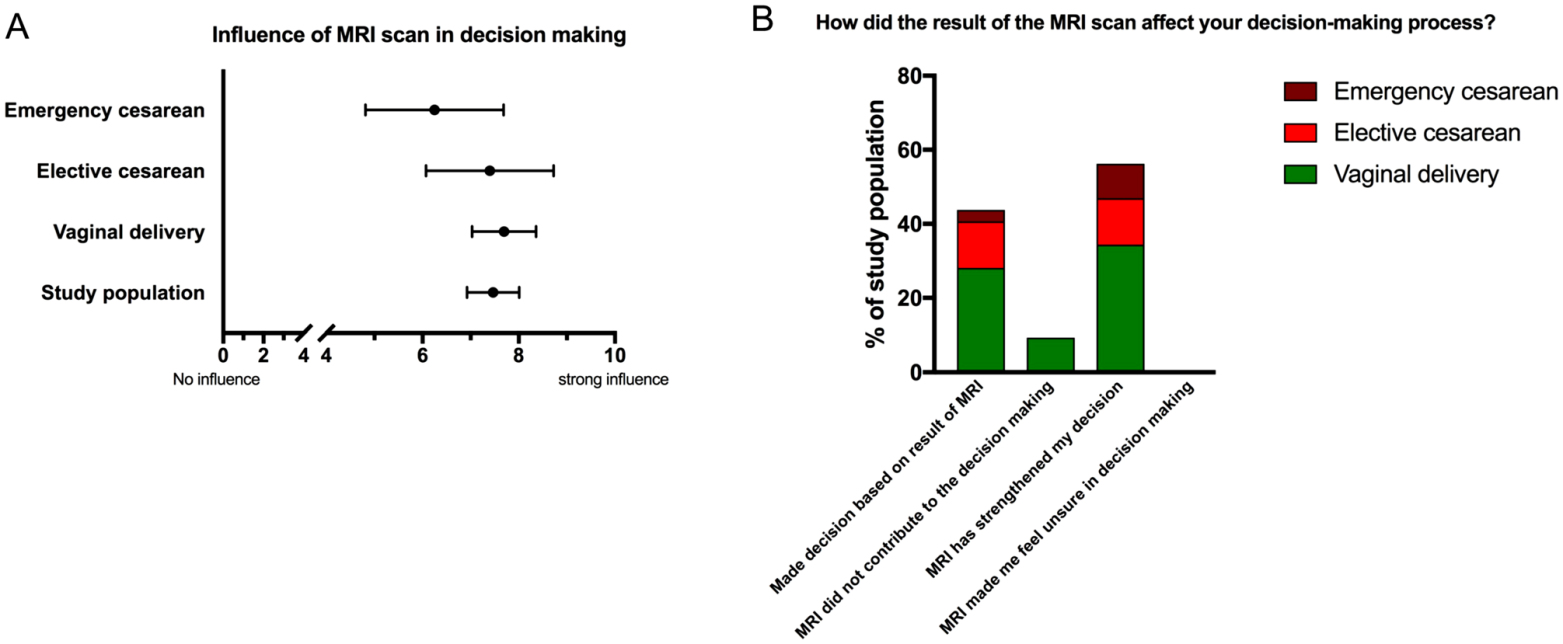
Influence of MRI in the decision-making process. Fraction of collective with vaginal delivery is shown in green, elective cesarean is shown in red and emergency cesarean is shown in brown

The participants of all groups were approximately equally strong influenced by MRI imaging regarding the process of decision-making as assessed by self-report (Fig. 4).

### Birth experience

Women were asked to provide details on their birth experiences. The MRI pelvimetry contributed a greater sense of security for all three subgroups. The elective cesarean section group and the group of those who delivered vaginally were approximately equally highly satisfied with their feeling of self-determination of the birth mode. Regarding safety feeling during birth, the average assessment of the study population was very high. Women who had emergency cesarean section felt less safe during birth.

**Fig. 5:**
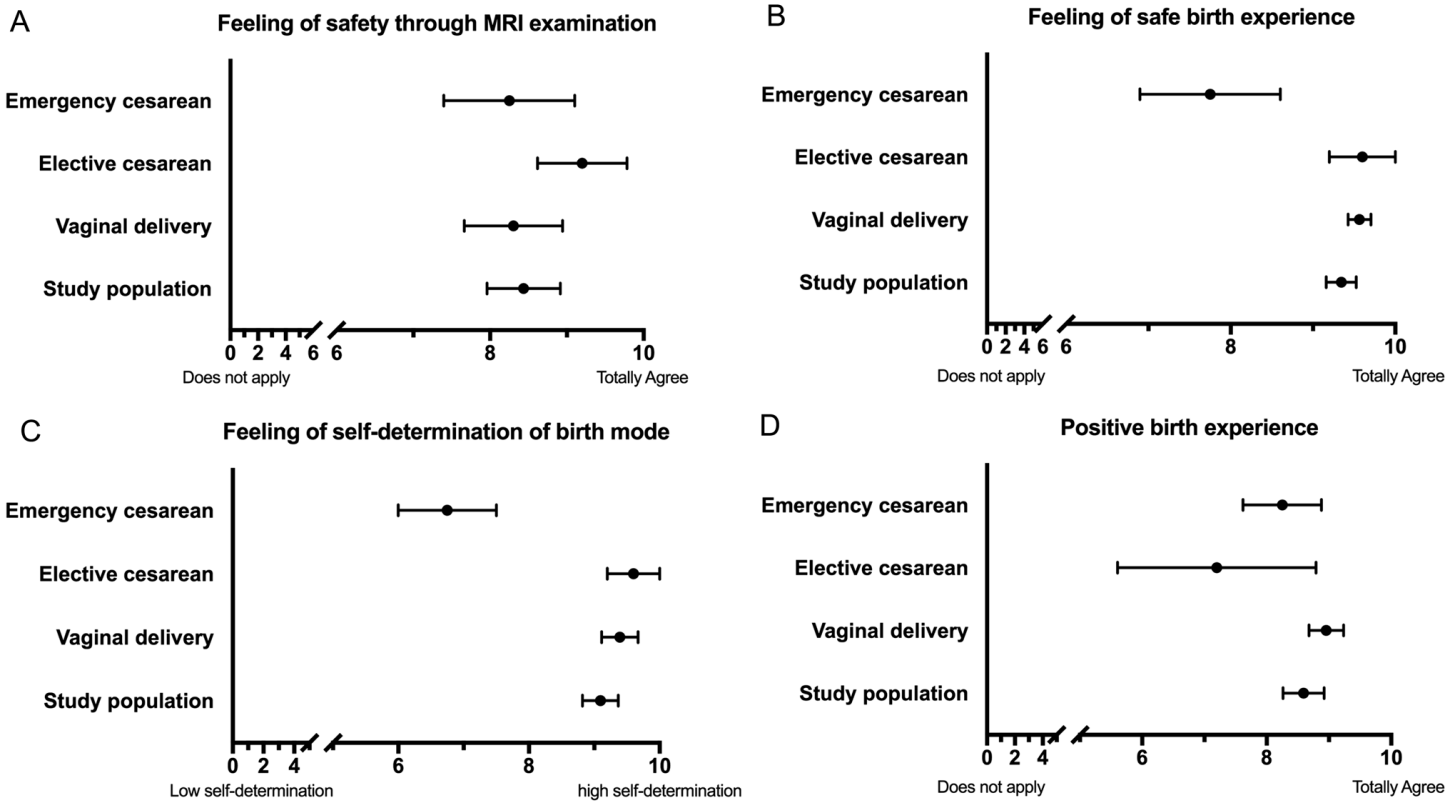
Feeling of safety through MRI examination, safe and positive birth experience and self-determination while giving birth

Overall, the study population had a very positive birth experience. The group of women who had delivered by elective cesarean showed a wider range in their assessment and appeared to perceive the experience more negative than the group of women who had a vaginal birth or emergency cesarean (Fig. 5). The maternal and fetal outcome is shown in Table 3.

**Table 3:** Maternal and fetal outcome

Characteristics	Study population	Vaginal delivery	Cesarean delivery	Elective cesarean	Emergency cesarean
Birth weight in gram, mean ± STD	3394 ± 390	3344 ± 360	3518 ± 453	3405 ± 536	3659 ± 342
Birth weight percentile, mean ± STD	41.5 ± 25.3	40.3 ± 24.9	50.6 ± 31.9	38.4 ± 34.8	65.8 ± 23.2
Head circumference in cm, mean ± STD	35.5 ± 1.4	35.5 ± 1.4	35.6 ± 1.2	35.5 ± 1.4	35.6 ± 1.3
Head circumference percentile, mean ± STD	62.5 ± 25.4	63.5 ± 24.5	60.6 ± 28.6	55.8 ± 31.6	66.5 ± 27.6
Arterial umbilical cord pH, mean ± STD	7.22 ± 0.08	7.20 ± 0.08	7.27 ± 0.07	7.25 ± 0.08	7.29 ± 0.04
Arterial umbilical cord base excess, mean ± STD	−6.1 ± 4.4	−7.5 ± 4.1	−2.7 ± 3.2	−2.5 ± 4.2	−2.9 ± 1.8
Arterial umbilical cord lactate, mean ± STD	5.6 ± 3.2	6.8 ± 3.1	3.1 ± 1.7	2.1 ± 0.4	4.1 ± 2.0
APGAR 1 min, mean ± STD	8.7 ± 1.1	8.6 ± 1.3	8.9 ± 0.3	8.8 ± 0.4	9.0 ± 0
APGAR 5 min, mean ± STD	9.8 ± 0.6	9.8 ± 0.7	9.9 ± 0.3	9.8 ± 0.4	10.0 ± 0
APGAR 10 min, mean ± STD	9.9 ± 0.2	9.9 ± 0.2	10.0 ± 0	10.0 ± 0	10.0 ± 0
Newborn stay at NICU, *N* (%)					
Yes	1 (3.1)	1 (4.3)	0 (0)	0 (0)	0 (0)
No	31 (96.9)	22 (0)	0 (0)	0 (0)	0 (0)
Newborn hospital stay in days, mean ± STD	2.5 ± 1.2	2.5 ± 1.3	2.8 ± 0.8	2.4 ± 0.9	3.3 ± 0.5
Mother hospital stay in days, mean ± STD	3.5 ± 1.4	3.4 ± 1.3	3.7 ± 1.6	2.8 ± 0.8	4.8 ± 1.7

## Discussion

Shared decision-making is a process for the individual development of a patient decision. This decision should be patient-centered, individualized, and considering the patient's current circumstances after discussing the benefits and risks of the available treatment options. The values and the priorities of the patient play a special role here [17].

Between the diagnosis of breech position and delivery, the women proceed through an emotional process that has to result in the acceptance of anticipation of a birth other than the one idealized. In addition, they have to decide on the mode of delivery. The majority of the study group did not know about MRI pelvimetry as an examination providing more information for assessing the success of vaginal delivery, but found it to be an important factor during decision-making regardless the later chosen birth mode, when asked retrospectively.

Most women in our study knew about the opportunity of vaginal breech delivery. International societies regard breech delivery as safe [18]. Long-term data show that planned cesarean section is not associated with a delay in neurodevelopment or a reduced risk of death compared to planned vaginal delivery [2, 19]. Nevertheless, in these women, at first there was uncertainty and a strong desire for the child to turn in cephalic position to avoid fetal complications. The information the pregnant women received immediately after the diagnosis of breech position was made or obtained independently afterward was unsatisfactory overall. To improve the quality of obstetric care, improved patient education and a greater focus on patient preferences could help eliminate uncertainty early on.

System factors and provider contribute to delivery decisions and are susceptible elements of intervention to achieve good delivery mode education and preparation [20].

The individual attitude of the obstetrician who carries out the educational work seems to have an influence on the choice of delivery mode of the mother. Studies showed that when women felt that their provider had a preference, they were more likely to choose the provider's preferred method of delivery [21, 22].

Patient preferences should be incorporated along with safety and medical effort, and providers have a duty to offer balanced advice that includes a realistic delineation of risks and benefits. This process can primarily help patients make medically appropriate decisions in line with their goals, which can create an appropriate population for spontaneous breech delivery.

In Germany, evidence from the Term Breech Trial has changed practice. The results led many providers and obstetricians in the clinics to offer the pregnant women an external cephalic version or a primary cesarean section. Given that most women have a preference for vaginal birth as we showed in this study, the MRI pelvimetry can help us to provide more information for assessing the success of vaginal delivery and strengthen the decision to give birth normally and provide security. Based on the additional information provided by the MRI pelvimetry and the usual information about existing studies and the experience of the clinic, the expectant mother can make an informed decision. This can increase her satisfaction with the birth experience and help to reduce anxiety and stress [23].

Overall, the study population had a very positive birth experience. The group of women who had delivered by elective cesarean showed a wider range in their assessment and appeared to perceive the experience more negative than the group of women who had a vaginal birth or emergency cesarean.

Women who underwent a planned cesarean were more likely to feel like they were not playing an active role during birth process [24], whereas successful vaginal delivery or the attempt at it leads to confidence in the power and capabilities of one´s own body, resulting in greater satisfaction from the overall birthing experience [25]. The rate of aborted vaginal breech deliveries (14.1%) was substantially lower in our study than in the Term Breech Trail (43.9%) [2]. The short-term neonatal outcome was not different between group of vaginal delivery, elective cesarean section, and emergency cesarean section. The complication rate of the women was also not different in the different delivery groups. These convincing results can be attributed to the great obstetric experience in our department as a specialized center for breech delivery. Furthermore, the preselection for the offer of spontaneous delivery from a breech position seems to be appropriate.

All women who could imagine a vaginal birth after being informed in detail, were offered an MRI scan. 50% of the respondents had not decided for a mode of delivery before having MRI pelvimetry. After imaging and information about the pelvic dimensions and the possibility of a vaginal birth, 80% of this subgroup decided to give birth vaginally. However, an MRI examination did not request an intended vaginal birth later. Rather, pregnant women should be given maximum information to choose their mode of delivery. 20% of the participants chose a primary cesarean section despite favorable conditions for a vaginal delivery. Nevertheless, in these cases, MRI seemed to be a useful examination tool in the decision-making process when choosing the optimal delivery method for this woman.

Over 40% of the collective described that they made a decision based on the result of MRI pelvimetry. None of the women felt to be insecure after having talked about the MRI results. The elective cesarean section group and the group of those who delivered vaginally were approximately equally highly satisfied with their feeling of self-determination of the birth mode. Our study shows that the MRI pelvimetry contributed a greater sense of security for all three subgroups.

The principle of shared decision-making plays a crucial role in obstetrics. In this special process, it is crucial that obstetricians have an appreciative and empathetic attitude toward their patients and at the same time continue to be the experts. They are anticipating possible anxieties and can encourage their patients to possible fewer injuring therapies. Studies indicate that insufficient shared decision-making increases feeling of loss of control, powerlessness, and helplessness. This can lead to a bad birth experience and an increase in postpartum depression [26].

Our study has some limitations. The collective examined here does not represent an average of the population. The women were mostly highly educated, native speakers, and very compliant. All of them have dealt intensively with mode of delivery and have taken advantage of more preventive examinations than the average collective by carrying out an MRI pelvimetry. Furthermore, the long-term outcomes of mother and child have not been studied.

It is very important to provide the patients with clear information when breech presentation occurs to initiate the process of shared decision-making regarding birth mode. Getting over a birth process empowers the women to a new expanded body experience. This has the great potential to increase their resilience if previously discussed procedures have met with good outcomes and expectations.

MRT pelvimetry measurements can be used as a predictor for a successful vaginal breech delivery. The additional information obtained from the MRI measurements can be used in the shared decision-making process to decide more easily on the mode of delivery while improving women's awareness and safety. Balanced education, rare and frequently adverse events of vaginal delivery and cesarean section, and patient expectations about labor processes must be taken into account.

## Data Availability

The datasets used and analyzed during the current study are available from the corresponding author on reasonable request.
